# Spontaneous Tumor Lysis Syndrome as Presenting Sign of Metastatic Prostate Cancer

**DOI:** 10.7759/cureus.3706

**Published:** 2018-12-08

**Authors:** Amy McGhee-Jez, Vivek Batra, Tara Sunder, Sanaa Rizk

**Affiliations:** 1 Oncology, Thomas Jefferson University Hospital, Philadelphia, USA; 2 Internal Medicine, Thomas Jefferson University Hospital, Philadelphia, USA; 3 Internal Medicine, Thomas Jefferson University Hospital, Philadelphia , USA; 4 Hematology, Thomas Jefferson University Hospital, Philadelphia, USA

**Keywords:** thrombotic microangiopathy, tumor lysis syndrome, thrombotic thrombocytopenic purpura, renal failure, prostate cancer

## Abstract

Spontaneous tumor lysis syndrome is an exceedingly rare manifestation of metastatic prostate cancer. It can masquerade as thrombotic thrombocytopenic purpura (TTP) or complement-mediated hemolytic uremic syndrome (HUS). These entities present with microangiopathic hemolytic anemia, thrombocytopenia, and renal failure, and improve with the initiation of plasma exchange and steroids. In situations where the laboratory data does not wholly validate the presumed diagnosis and clinical and laboratory deterioration occurs in spite of appropriate treatment, it is necessary to expand the differential diagnosis and investigation. In this case, worsening renal function, cytopenias, lactate dehydrogenase, and uric acid in the setting of proper treatment for TTP and complement-mediated HUS prompted additional analysis. This workup revealed bone marrow infiltration by metastatic prostate cancer complicated by tumor lysis syndrome.

## Introduction

Tumor lysis syndrome (TLS) is characterized by hyperuricemia, hyperkalemia, hyperphosphatemia, and concurrent hypocalcemia [1]. It is a frequent cause of renal failure in hematologic malignancies and rarely observed in patients with solid tumors, particularly those with metastatic prostate cancer [1]. We highlight the diagnostic workup and treatment in a patient who was incorrectly diagnosed with thrombotic thrombocytopenic purpura (TTP) and subsequently found to have metastatic prostate cancer complicated by TLS.

## Case presentation

A 49-year-old African-American male with a past medical history of sickle cell trait was transferred to our tertiary care hospital from a local community hospital. He initially presented to the outside hospital with one week of fatigue, arthralgias, and myalgias. Given anemia (hemoglobin 11 g/dL), thrombocytopenia (platelet count 46,000 per microliter), acute renal failure (creatinine 1.33 mg/dL, elevated from a normal baseline), elevated lactate dehydrogenase (LDH; 968 IU/L), decreased haptoglobin (15 mg/dL), and a peripheral blood smear showing one to two schistocytes per high power field (HPF), he was presumed to have TTP. An "a disintegrin and metalloproteinase with a thrombospondin type one motif, member 13" (ADAMTS13) was appropriately sent and pending at time of transfer. Additionally, his white blood cell count was 4.1 per microliter, potassium 4.1 mmol/L, phosphate 6.6 mg/dL, calcium 9.8 mg/dL, and liver function tests showed elevated bilirubin of 2 mg/dL. He was empirically started on 1 mg/kg prednisone and daily plasma exchange (one plasma volume per day). Given lack of improvement with these interventions and three days of plasma exchange (PLEX), he was referred to our hospital.

Upon presentation to the initial hospital, his review of systems was positive for intermittent rigors, constipation, and low back pain. He denied unintentional weight loss, fever, bleeding or bruising, dyspnea, or urinary symptoms. He used occasional ethanol, but denied any smoking or drug use history. He denied recent travel or risk factors for human immunodeficiency virus (HIV). His family history was non-contributory.

**Figure 1:**
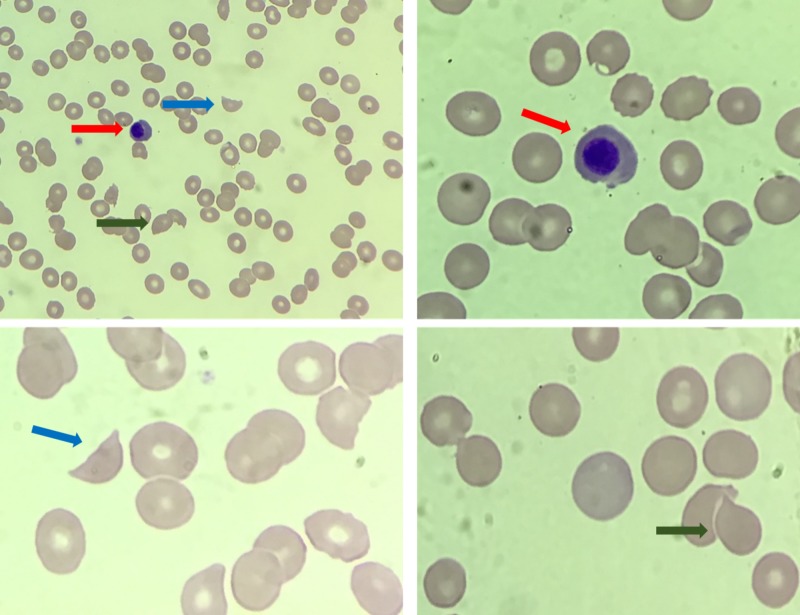
Sample pictures from patient’s peripheral blood smear showing few schistocytes, nucleated red blood cells, and tear drops. Red arrows show nucleated red blood cells. Blue arrows show schistocytes. Green arrows show tear drops.

On examination, he was an ill-appearing thin tall male with abdominal tenderness and diffuse pain on palpation of the lower back, shoulders, and hips. His pertinent laboratory data at our hospital after four days of prednisone and three days of daily PLEX included hemoglobin of 7.6 g/dL, mean corpuscular volume (MCV) 86 fL, white blood cell count 3.3 per microliter, platelet count 25,000 per microliter, and inappropriately low reticulocyte percentage of 0.9% (absolute reticulocyte count of 22 per microliter). His renal function was rapidly deteriorating (creatinine 5.4 mg/dL). Haptoglobin improved to 60 mg/dL while his LDH rose to 4445 IU/L. Ferritin was significantly elevated (31,863 ng/mL) with elevated uric acid (11.7 mg/dL). Urinalysis was notable for proteinuria and hematuria without casts. Peripheral smear at our institution revealed occasional schistocytes (one to two per HPF) with few nucleated red cells and teardrops (Figure 1). Given hypoproliferative anemia and peripheral smear with teardrops and nucleated red blood cells, a bone marrow biopsy was performed to rule out infiltrative disease.

While results of the bone marrow biopsy were pending, the patient’s renal function, anemia, and thrombocytopenia continued to worsen in the setting of daily PLEX and steroids. He became anuric and required initiation of hemodialysis. At that time, it was decided to stop PLEX and administer a trial of eculizumab as complement-mediated hemolytic uremic syndrome (HUS) was rising on the differential. ADAMTS13 results became available showing appropriate activity of 151%, supporting the decision to stop PLEX. The patient also underwent imaging of the chest, abdomen, and pelvis to evaluate for occult malignancy. This was notable for abnormal diffuse mottled and moth-eaten attenuation of the spine suggestive of a diffuse marrow process, but no evidence of malignancy.

Final pathology of the bone marrow biopsy revealed metastatic prostate adenocarcinoma with neuroendocrine differentiation with nearly absent trilineage hematopoiesis and greater than 90% necrosis of the marrow space. The etiology of the patient’s renal failure, cytopenias, elevated uric acid, and elevated LDH was thus favored to be tumor lysis syndrome from metastatic prostate adenocarcinoma which may have been precipitated spontaneously or with the administration of high dose steroids. Of note, the prostate specific antigen (PSA) was elevated at 24.9 ng/ml.

At this point, he was initiated on allopurinol and supported with transfusions and hemodialysis. Steroids were tapered. He started palliative chemotherapy with carboplatin and etoposide on day 12 of hospitalization.

## Discussion

Tumor lysis syndrome is infrequently seen in the management of solid tumors [1]. More often, it is a complication of hematologic malignancies and is characterized by elevated urate, LDH, phosphate, and potassium with concomitantly low calcium which can lead to cardiac, neurologic, and renal dysfunction [1]. It is often a consequence of cytotoxic chemotherapy, but can occur spontaneously in bulky, highly proliferative malignancies [1]. In a 2014 review by Mirrakhimov et al., the authors documented 112 cases of tumor lysis syndrome in solid tumor patients. Only five of these were patients with prostate cancer [1]. Further, the mortality rate of TLS in this patient population is significantly higher than that reported in the hematologic malignancy population [2].

In our review of the literature, we found nine cases of TLS occurring in patients with prostate cancer [2-10]. The majority of these cases involved the development of TLS after initiation of cancer-directed therapy [2-5, 8, 9]. Specifically, there were three cases of TLS after cytotoxic chemotherapy with either docetaxel or paclitaxel and two cases after androgen deprivation therapy (ADT), both with and without radiation therapy [2-5, 8]. There was also one documented case of TLS after radiation therapy alone [9]. Notably, there are only two documented cases of TLS occurring spontaneously in patients with treatment-naive metastatic prostate adenocarcinoma and one case of spontaneous TLS after documented progression of disease while on ADT [6, 7, 10]. Our patient may be the third known case of TLS in treatment-naive metastatic prostate cancer and the only case of TLS in metastatic prostate adenocarcinoma with neuroendocrine differentiation. It is still unclear if the patient presented here developed autolysis or if the high-dose prednisone he received for presumed TTP initiated tumor lysis. Based upon the extensive necrosis in his bone marrow and worsening renal function and LDH with initiation of steroids, we tend to favor TLS induced by steroids.

In addition to the rarity of spontaneous TLS in metastatic prostate cancer, our patient’s case is also distinct in that he had no evidence of a metastatic malignancy on his initial workup. In the nine cases listed above, all patients had evidence of widely metastatic disease, often with bone and liver involvement that was easily seen on imaging. Our patient’s imaging, however, did not show evidence of malignancy other than an indeterminate abnormal bone marrow signal. Similarly, the PSA in the majority of these patient cases was significantly elevated while our patient’s PSA was only moderately high at 24.9 ng/mL.

Lastly, as mentioned previously, the mortality rate of TLS in solid malignancies is quite high. Consistent with this, the mortality rate in the patient cases we reviewed was high as well. All patients described died prior to publication. Specifically, patients who developed TLS secondary to treatment died within 40 hours to two months of therapy initiation, with the majority expiring within 10 days [2-5, 8, 9]. Those patients who developed spontaneous TLS died within six weeks of TLS recognition [6, 7, 10]. Fortunately, our patient has done well thus far. He was treated with cycle one of carboplatin and etoposide, both renally dosed, as an inpatient and tolerated this well. This regimen was chosen based upon the neuroendocrine features of his prostate cancer. At the time of this publication, he has completed four cycles of chemotherapy as an outpatient and is dialysis independent. Unfortunately, subsequent bone marrow biopsy after treatment showed persistent disease six months after his initial diagnosis, so he is currently starting immunotherapy with ipilimumab and nivolumab.

Teaching points extracted from prior case reports include recognition and prevention of TLS in patients with bulky, aggressive metastatic disease despite the rarity of its occurrence in prostate cancer [2, 6-8]. This can be achieved by checking TLS labs, especially LDH and urate, prior to the initiation of cancer-directed therapy. Appropriate prophylaxis can then be instituted with intravenous fluids, allopurinol, and close surveillance. While these are vital points to keep in mind, they did not apply to the patient presented here, making this case an important contribution to the current literature. While the original concern at presentation was for TTP given acute renal failure, anemia, thrombocytopenia, and suspected hemolysis, there are details to note which help distinguish TLS from TTP in this patient. His initial peripheral smear showed one to two schistocytes per HPF (Figure 1) and the first documented haptoglobin was 15 mg/dL. If this had been TTP, one would expect many more schistocytes and an undetectable haptoglobin [11]. Additionally, his peripheral smear also showed nucleated red blood cells and tear drop forms in the setting of a peripheral reticulocytopenia and pancytopenia, suggesting an intrinsic bone marrow process. This was also suggested on his computed tomography (CT) scan. He appropriately underwent urgent plasma exchange and steroid administration, but subsequent laboratory evaluation demonstrated worsening renal function and cytopenias, as well as rising LDH, despite a rising haptoglobin. This further supported the notion that the patient did not have MAHA (microangiopathic hemolytic anemia). He appropriately underwent a bone marrow biopsy, which confirmed the diagnosis of metastatic prostate adenocarcinoma with neuroendocrine differentiation. It is likely that his marrow was nearly replaced with prostate cancer and the introduction of high dose steroids precipitated TLS causing his lab abnormalities and the extensive necrosis seen on bone marrow biopsy. While it would be very difficult to anticipate this clinical scenario in future patients, it is important to note the details of the patient’s peripheral smear, reticulocytopenia, indeterminate hemolysis labs, and lack of response to plasma exchange in order to expand the differential diagnosis and institute a prompt investigation for an underlying malignancy complicated by the presence of TLS.

## Conclusions

This case highlights TLS as a cause of acute renal failure and emphasizes the necessity of careful inspection of the laboratory data and peripheral smear in working through a differential diagnosis of thrombocytopenia, anemia, and renal failure. While recognition of potential TTP and rapid initiation of PLEX remain of utmost importance, it is also necessary to expand the differential when pheresis and steroids fail to help. Rising uric acid, LDH, and creatinine in the setting of haptoglobin normalization suggests a process other than hemolysis, namely TLS. Lastly, this highlights a rare case of spontaneous tumor lysis syndrome from metastatic prostate cancer.
